# Excision of Scirrhous Rectum

**Published:** 1831

**Authors:** 


### Excision of Scirrhous Rectum.

Our readers are well aware that M. Lisfranc is in the habit of excising the lower extremity of the rectum for what is called in France cancer of that gut. Our readers are probably aware also, that the term in question is not restricted in that country to the same malignant class of disease, to which it is limited by us; and consequently that in many cases the operation is performed for affections not essentially malignant. This being premised, we may mention some circumstances respecting excision of the rectum, which are not undeserving of attention.

The peritoneum descends along the front of the rectum to six inches from its extremity in woman, to four inches from the same in man. By means of an ovoid incision in the skin around the anus, the rectum can readily be drawn out behind, and any kind of instrument may be applied to it; there exists a second sphincter above the first. M. Lisfranc has removed as much as three inches and a half of the rectum, and he recommends the operation whenever the fore-finger can reach beyond the upper margin of the disease, and when the cellular texture external to the gut is sound. The operator must bear in mind that the antero-posterior diameter of the perineum is generally one inch, the distance of the anus from the coccyx eighteen lines, and that between the anus and the base of the same bone two inches; that considerable portions of the rectum may be removed laterally and posteriorly without wounding the vagina in woman or the urethra in man; and finally, that hæmorrhage may always be arrested by pressure or by ligatures. In the performance of the operation the patient is to be placed as in the lateral operation for lithotomy—two semilunar incisions are to be made around the anus—and the rectum to be insulated in its inferior extremity, drawn down by the fore-finger introduced into its cavity, and cut off by means of scissors. After the cure, the fæces are sometimes voided in the usual manner, sometimes a *bourrelet* is formed internally and takes the place of the sphincter, sometimes there is incontinence of liquid fæces, and sometimes the patient is obliged to stuff the rectum with lint. Out of nine cases of operation related by M. Lisfranc, six ended favourably, three fatally.

